# Replaying the Tape of Life: Quantification of the Predictability of Evolution

**DOI:** 10.3389/fgene.2012.00246

**Published:** 2012-11-26

**Authors:** Alexander E. Lobkovsky, Eugene V. Koonin

**Affiliations:** ^1^National Center for Biotechnology Information, National Library of Medicine, National Institutes of HealthBethesda, MD, USA

**Keywords:** evolutionary trajectory, predictability of evolution, fitness landscape, divergence of trajectories

## Abstract

The question whether adaptation follows a deterministic route largely prescribed by the environment or can proceed along a large number of alternative trajectories has engaged extensive research over the recent years. Experimental evolution studies enabled by advances in high throughput techniques for genome sequencing and manipulation, along with increasingly detailed mathematical modeling of fitness landscapes, are beginning to allow quantitative exploration of the repeatability of evolutionary trajectories. It is becoming clear that evolutionary trajectories in static correlated fitness landscapes are substantially non-random but the relative contributions of determinism and stochasticity in the evolution of specific phenotypes strongly depend on the specific conditions, particularly the magnitude of the selective pressure and the number of available beneficial mutations.

## 1 Measuring Evolutionary Repeatability

The great evolutionist Stephen Jay Gould often employed the metaphor “replaying life’s tape” to emphasize the preeminent role of contingency in the evolutionary process (Gould, 1990). In Gould’s view, the outcome of this Gedanken experiment would have been dramatically different from the actually observed course of events because evolution is essentially a stochastic phenomenon whereby trajectories that start infinitely close to each other soon diverge because the divergence is exponential. The actual evolutionary trajectory is fundamentally unpredictable because the survival of the fittest could occur along a great number of forking paths. Until recently, the relative contributions of determinism and stochasticity in the evolutionary process had been a problem that, however fascinating, could not be addressed by direct means. The well-established (near) neutrality of many (probably a substantial majority) of the fixed mutations (Kimura, 1983, 1986; Koonin and Wolf, 2010; Nei et al., 2010) as well as the fact that many biological functions are mediated by unrelated genes in different organisms (non-orthologous gene displacement; Koonin, 2005; Omelchenko et al., 2010) indicated that the stochastic component is substantial but the quantification of the (un)predictability of evolution remained elusive. The precise definition of predictability when applied to trajectories has not been settled on yet. Our purpose here is to promote the notion of the mean path divergence as a quantitative measure of predictability when applied to a statistical ensemble of trajectories that share the endpoints.

Over the last decade, the advances of technologies for rapid DNA sequencing and mutagenesis have enabled the launch of the experimental evolution field. Evolutionary experiments under controlled laboratory conditions allow researchers, at least in principle, to directly address the problem of the predictability of evolutionary processes. Prediction of the actual trajectory of evolution is a daunting task that requires a complete and detailed understanding of the mutational effects on phenotype (Loewe, 2009; Papp et al., 2011). A narrower form of Gould’s question is closely related to this problem: does adaptation that leads to an *a priori* known phenotype follow a quantifiably repeatable route? In other words, if the starting and ending phenotypes are known, how similar in a quantitative sense are the trajectories that lead from one to the other? Answers to both questions can be given on different genomic scales. For example, one might be interested in predicting the groups of genes that are likely to be involved in adaptation to a particular challenge. A higher resolution answer would enumerate specific genes involved and whether these genes are duplicated, (in)activated, or mutated. The nucleotide resolution answer to the question of repeatability must quantify the individual and combined effects of all mutations involved in adaptation. Given the exponential increase in the number of combinations with the number of mutations, even with next generation sequencing it is possible to obtain the nucleotide resolution answer only in cases where there are only a dozen or fewer important mutations.

A direct approach to quantifying evolutionary predictability requires massively parallel, carefully controlled, and fully characterized evolution experiments. Results from a substantial number of recent experiments allow preliminary conclusions on the question of evolutionary predictability to be formulated. To summarize, there is significant parallelism in adaptation on the level of functional groups and operons; these changes can be effected through a number of alternative mutations although there exist particularly important mutations that are required (Nakatsu et al., 1998; Wichman et al., 1999; Elena and Lenski, 2003; Pelosi et al., 2006; Blount et al., 2008; Gresham et al., 2008; Kao and Sherlock, 2008; Dickins and Nekrutenko, 2009; Cooper and Lenski, 2010; Crozat et al., 2010; Lang et al., 2011; Papp et al., 2011; Meyer et al., 2012). An important result at the nucleotide level is that many adaptive mutations require one or several compensatory mutations to manifest their full benefit or even to provide any benefit at all, so sign epistasis is an essential aspect of evolution that substantially constrains the evolutionary process (Weinreich et al., 2005; Davis et al., 2009; Brown et al., 2010).

Although important insights have been obtained via parallel evolution experiments, they present significant technical challenges that are only now becoming addressable. Achieving statistical significance in quantifying predictability requires characterization of numerous clones from a large number of evolving populations. Strict uniformity of the experimental conditions over time and among the evolving populations is essential as well because there is evidence that a fluctuating environment leads to a greater diversity of adaptive trajectories (Cooper and Lenski, 2010). Obtaining pertinent information on the predictability of evolution requires thorough understanding of the complex clonal interference dynamics that dominates chemostat experiments with large effective population sizes (Gresham et al., 2008; Kao and Sherlock, 2008; Lang et al., 2011; Miller et al., 2011). The tentative conclusion that emerges is that understanding the extent and the nature of the genetic variation, which requires extensive sequencing in a population, is paramount to predicting the evolutionary trajectory (Lang et al., 2011). Because of the technical challenges, most experiments either focus on viruses (Wichman et al., 1999, 2005; Dickins and Nekrutenko, 2009; Rokyta et al., 2009; da Silva et al., 2010; Miller et al., 2011; Meyer et al., 2012) or study a situation in which only a few genetic loci are implicated in the adaptive process (Kellam et al., 1994; Medeiros, 1997; Weinreich et al., 2006; O’Maille et al., 2008; Brown et al., 2010; da Silva et al., 2010; Salverda et al., 2011; Toprak et al., 2012). However, thanks to the advent of next generation sequencing (Kircher and Kelso, 2010; Metzker, 2010), several attempts have been recently made to conduct precisely controlled parallel evolution experiments in tandem with high precision full genome sequencing of multiple bacterial or yeast clones (see details below).

A complementary approach to quantifying evolutionary predictability is based on the idea of a fitness landscape that was originally introduced in the seminal work of Sewall Wright as a metaphor and an illustration of evolutionary paths from low fitness to high fitness genotypes (Wright, 1932, 1988). A key simplifying assumption behind the concept of a fitness landscape is that in a fixed environment there is a functional relationship between the genome of an organism and its growth rate, i.e., its Malthusian fitness (Gavrilets, 2004). The concept of a fitness landscape has been influential in shaping the discussion in many areas of research on molecular evolution and considerable effort has been expended in understanding the properties of empirical landscapes as well as characterizing model landscapes (Kauffman and Levin, 1987; Kauffman and Weinberger, 1989; Aita et al., 2000; Lunzer et al., 2005; Hayashi et al., 2006; Miller et al., 2006, 2011; Beerenwinkel et al., 2007; Poelwijk et al., 2007, 2011; O’Maille et al., 2008; Kogenaru et al., 2009; Kryazhimskiy et al., 2009; Carneiro and Hartl, 2010; Dawid et al., 2010; Novais et al., 2010; Costanzo and Hartl, 2011; Costanzo et al., 2011; Franke et al., 2011; Lobkovsky et al., 2011).

Here we review the findings of parallel evolution experiments specifically focusing on the conclusions regarding predictability. We proceed to discuss experiments that completely reconstruct small regions of fitness landscapes and theoretical efforts to analyze the degree of repeatability of evolution on these landscapes. We then introduce the concept of path divergence and use it to further quantify repeatability of mutational trajectories in empirical and model fitness landscapes.

## 2 Parallel Evolution Experiments

Parallel evolution experiments typically choose one of several common model systems: bacteriophages, *Escherichia coli* (and other well-characterized, easily cultivable bacteria), yeast and others, and subject several, initially identical wild type populations to an environmental challenge such as nutritional limitation, antibiotic exposure, or elevated temperature. Successful clones from each population are sequenced and the genetic basis of their phenotypic adaptation is sought.

In one of the earliest parallel evolution experiments, Wichman et al. (1999) studied two parallel bacteriophage lines under strong selection and found that each underwent over a dozen adaptive nucleotide substitutions after over a thousand population doublings. Although, strikingly, half of the substitutions were shared between the two lines, the order of their appearance differed between the two lines suggesting that there was minimal if any epistasis between the common mutations. In general, in the absence of epistasis, i.e., the situation where the combined fitness effect of any number of mutations is simply the sum of their individual effects, beneficial mutations can appear in any order while improving fitness at every step. The mutations that were unique to each of the two bacteriophage lines in the experiments of Wichman et al. (1999) might represent compensatory changes that are drawn from a much larger pool of possibilities. Another study of 7 closely related bacteriophages (Rokyta et al., 2009) also found overwhelming parallel evolution on the nucleotide level even among phages that differed by as much as 7% of the genome sequence. Several other studies of bacteriophage adaptation reported similar results (Wichman et al., 2005; Dickins and Nekrutenko, 2009; Miller et al., 2011; Meyer et al., 2012).

Human immunodeficiency virus adapting to an alternative chemokine receptor evolves via a set of 7 mutations (Boucher et al., 1992; Kellam et al., 1994; da Silva et al., 2010). da Silva et al. (2010) assayed the fitness of a large number of mutants that contained combinations of these 7 mutants to discover strong and complex epistatic relationships between substitutions. The authors computed the probabilities of observing different adaptive trajectories and found that, although no single dominant trajectory existed, a random trajectory hypothesis could be rejected.

Evolution of drug resistance in a more complex organism such as *Plasmodium falciparum* (Lozovsky et al., 2009; Brown et al., 2010; Costanzo and Hartl, 2011) typically involves a small number of mutations in one or several loci. Traditionally, minimum inhibitory concentration (MIC or IC50) of mutants is measured rather than the growth rate in a fixed drug environment. Because the growth rate is a monotonic function of the drug concentration, MIC can be used as a proxy for fitness. The analysis of adaptive trajectories that involve 6 mutations in the Dihydrofolate Reductase (DHFR) locus (Costanzo et al., 2011) has shown that, although there were 3 high resistance alleles, one of these was reached substantially more often, presumably because of the greater availability of monotonic fitness trajectories leading to this particular genotype. In addition, when all possible trajectories are considered, only a small fraction are monotonic in fitness and hence accessible to evolution under strong selection.

Bacteria can be subjected to a broader range of challenges. In one long term experiment, 18 lines of *Ralstonia* grown for over a thousand generations on an alternative carbon source (Nakatsu et al., 1998) exhibited parallel duplications of a common plasmid segment and several common deletions. Blount et al. (2008) have shown that *Escherichia coli* grown in a citrate-rich but glucose-limited medium required several potentiating mutations that did not themselves confer a fitness advantage. Selectively neutral potentiating mutations could occur in any order and thus the adaptive trajectory is not repeatable at the nucleotide level. However, when focus is shifted to the level of genes and operons (Woods et al., 2006), or gene expression profiles (Pelosi et al., 2006), extensive parallelism is uncovered. Ostrowski et al. (2008) studied *E. coli* adapting to a glucose environment and identified 21 mutations (18 unique) in five alternative loci/gene regions. Although all mutants fared equally well on glucose, fitness on alternative nutrients varied greatly, with the substitutions in the same locus clustering together phenotypically. Whole-genome re-sequencing of 5 parallel populations of *E. coli* grown in glycerol (Herring et al., 2006) revealed extensive parallelism at the level of genes. Although not exactly shared between populations, mutations were found in the same regions of the affected proteins.

Evolution of resistance to a large class of antibiotics involves several mutations in the TEM-1 β-lactamase locus (Medeiros, 1997). Salverda et al. (2011) subjected 12 replicates of *E. coli* to *in vitro* mutagenesis and selection in the presence of cefotaxime and found a high degree of repeatability in the order of appearance of the adaptive mutations. Remarkably, the first fixed mutation was particularly important in determining the entire trajectory. Toprak et al. (2012) developed a device that dynamically adjusts the concentration of antibiotic in an evolving bacterial population to keep the growth rate constant. Whole-genome sequencing revealed that resistance of *E. coli* to chloramphenicol and doxycycline involved mutations in about a dozen genes involved in translation, transcription, and transport. The authors hypothesized that a smoothly increasing fitness and a weak albeit significant parallelism at the nucleotide level were due to a large target for adaptation (that is, adaptation could involve a variety of genes that provided resistance at different stages of the antibiotic action and delivery to the cell). In contrast, mutations involved in adaptation to trimethoprim were strictly localized to the DHFR gene and its promoter. In this case, the small size of the mutational target and presumably small number of available beneficial mutations resulted in the step-wise fitness evolution. In addition, extremely high parallelism was observed in the order of adaptive mutations (Toprak et al., 2012).

Evolution of *E. coli* in an elevated temperature environment (Tenaillon et al., 2012) was found to be overwhelmingly adaptive (80% of the fixed mutations were estimated to be beneficial), leading to an almost twofold increase in fitness. Although two major pathways of high temperature adaptation were identified (one centering around the RNA polymerase, the other around the termination factor ρ), mutations tended to affect a small number of genes or functional units. However, because little parallelism was observed on the nucleotide level, the potential number of mutations beneficial in a high temperature environment appears to be large.

In eukaryotes, only a few parallel evolution experiments have been reported, and the results are more difficult to interpret. Yeast evolved under limited glucose conditions (Kao and Sherlock, 2008) exhibited a broad spectrum of adaptive mutations in a number of loci although the amplification of the *HXT6/HXT7* locus (a tandem array of genes encoding glucose transporters) was found in two out of the 5 studied lines. A detailed follow-up on this work has revealed a rugged fitness landscape dominated by reciprocal sign epistasis (Kvitek and Sherlock, 2011). Another, more extensive study of nutrient limitation effects in yeast (Gresham et al., 2008) identified diverse pathways of adaptation to glucose and phosphate limitation. However, sulfate limitation adaptation proceeded mainly via the amplification of the *SUL1* locus.

A remarkable case of convergent, apparently deterministic evolution, albeit coming from comparative rather than experimental studies, is that of the *PEPC* gene involved in the C^4^ photosynthesis (Christin et al., 2007; Besnard et al., 2009). A phylogenetic reconstruction of the *PEPC* evolution revealed that, although it has been recruited independently several times, 5 sites under positive selection underwent identical changes. These results recapitulate and expand the classic work of Stewart, Wilson, and colleagues that revealed 7 identical mutations occurring in parallel in the genes coding for lysozymes in cows and langur monkeys that are the only mammals adapted to cellulose fermentation in the foregut (Stewart et al., 1987; Swanson et al., 1991; Messier and Stewart, 1997).

To summarize, parallel evolution experiments find a broadly varying degree of repeatability of evolution. When the number of mutations implicated in adaptation is small (a few dozens at most) and/or there exists substantial epistasis between individual mutations, considerable degree of parallelism is observed on the nucleotide level although even in these cases there is usually a number of alternative beneficial mutations. Typically, substantial parallelism is observed at the more coarse-grained level of genes and operons although even at this level several alternative pathways to adaptation frequently exist.

## 3 Predictability of Evolution on Empirical Landscapes

Because of the technical challenges still involved in conducting parallel evolution experiments, their conclusions, however important, remain largely qualitative. An alternative approach with a promise for a quantitative understanding of the evolution repeatability is based on the direct measurement and analysis of fitness landscapes. The data are beginning to shed light on questions pertaining to the degree of epistasis and its variation across the landscape (Weinreich et al., 2005; Beerenwinkel et al., 2007; Martin et al., 2007; O’Maille et al., 2008; Davis et al., 2009; Kogenaru et al., 2009; da Silva et al., 2010; Dawid et al., 2010; Lunzer et al., 2010; Poelwijk et al., 2011; Salverda et al., 2011; Tenaillon et al., 2012), the distribution of fitness effects and its dependence on the genetic background (Aita et al., 2000; Lunzer et al., 2005; Martin et al., 2007; Davis et al., 2009; Lang et al., 2011; Miller et al., 2011), or the strength of correlations between mutations (Miller et al., 2006; Beerenwinkel et al., 2007; Knight et al., 2009; Rokyta et al., 2009). Here we focus on landscape features relevant to the questions of accessibility of high peaks and predictability of evolutionary trajectories. We also summarize the efforts to construct realistic model landscapes and compare them to empirical landscapes in order to extract model parameters and quantify the relationship between the measures of landscape ruggedness and the metrics of accessibility and predictability.

There are two complementary approaches to sampling the vast sequence space. The exhaustive sampling approach measures the fitness of a library of mutants that harbor all possible combinations of several important substitutions (Weinreich et al., 2006; O’Maille et al., 2008; Reetz and Sanchis, 2008; Novais et al., 2010; Franke et al., 2011). As pointed out by Franke et al. (2011), the mere fact that the chosen substitutions are deemed “important” (on the basis of preliminary experiments that detect these substitutions in the adapted organisms) and so only the small region of the landscape that harbors these substitutions is sampled exhaustively biases the explored portion of the landscape. The ruggedness of the explored part of the fitness landscape is likely to be exaggerated compared to the complete landscape. The key advantage of exhaustive sampling of a small part of the landscape is that measures of accessibility and predictability can be computed directly.

An alternative to an exhaustive study of a small region of the landscape is to take a broad but sparse sample of the landscape and rely on models to infer the complete landscape properties. Usually, a library of random mutants is constructed using error-prone PCR or a similar method. Measuring the fitness of the mutants of a highly fit sequence provides a glimpse into the properties of the landscape near a peak. The results of note include the confirmation of significant sign epistasis near a fitness peak and the observation that local deviations from additivity are derived from a nearly normal distribution (Weinreich et al., 2005; Beerenwinkel et al., 2007; Martin et al., 2007; Lunzer et al., 2010). If, alternatively, the process is started from a low fitness sequence, adaptive trajectories can be probed via repeated rounds of random mutagenesis and purifying selection (Bershtein et al., 2006; Miller et al., 2006; Romero and Arnold, 2009; Tracewell and Arnold, 2009). During adaptation mean fitness grows with each generation and eventually stagnates at a suboptimal plateau. The characteristics of the growth as well as the dependence of the plateau height on the library size can be used to classify fitness landscapes in a class of models introduced by Kryazhimskiy et al. (2009). A quantitative comparison to the *NK* model of random epistatic landscapes can even yield estimates of the model parameters (Kauffman and Weinberger, 1989; Hayashi et al., 2006).

To summarize, notable success has been achieved in comparing sparse fitness data to landscape models. However, simple models fail to account for all observed features of the landscapes and evolutionary trajectories whereas increasingly complex models (e.g., Rowe et al., 2010) tend to obscure interpretation of the data. It seems likely that the massively multidimensional nature of landscapes (Gavrilets, 2004) precludes a simple universal model applicable to all situations and custom models will need to be designed for each scenario.

**Figure 1:**
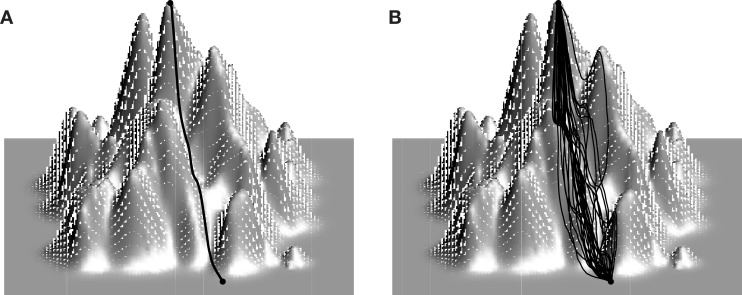
**Mutation rate and evolutionary trajectories on fitness landscapes**. When mutations are rare **(A)**, the population is nearly homogeneous and mutations are fixed sequentially via sweeps. Evolution can therefore be represented by a single path on the fitness landscape. Several distinct clones coexist at any given time in the population when mutation rate is high. **(B)** If each clone is allotted its own trajectory, the evolution of the population can be represented by a bundle of trajectories which split and terminate on the way to the summit. At any given time the population can be represented by a probability density function in sequence space.

**Figure 2:**
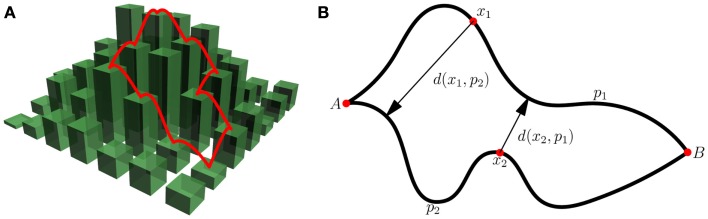
**Divergence of evolutionary paths on fitness landscapes**. **(A)** Demonstrates monotonic paths on a fitness landscape. Each path’s probability of occurrence in the SSWM limit is the inverse product of the number of “up” steps at each point along the path. The ensemble path divergence of the “ensemble” consisting of only these two paths would be the product of their probabilities of occurrence and the inter-path distance. **(B)** Illustrates the calculation of the inter-path distance *d*(*p*_1_, *p*_2_). For every point *x*_1_ ∈ *p*_1_ we find the closest point on *p*_2_ and record the distance *d*(*x*_1_, *p*_2_). We perform the same operation for points on *p*_2_. The distance is then *d*(*p*_1_, *p*_2_) = (∑_*p*1_*d*(*x*_1_, *p*_2_) + ∑_*p*2_*d*(*x*_2_, *p*_1_))/(length(*p*_1_) + length(*p*_2_)).

The fitness landscape is only one of the factors that determine whether evolution is predictable. The evolutionary dynamics controlled by the mutation rate and the strength of selection, which itself depends on the effective population size, are of major importance as well (Figure 1). When the mutation rate is low, mutations are either fixed or eliminated before another mutation arises. In this low mutation limit, the dynamics are composed of sweeps and the evolution of a population can be represented by a single trajectory on the fitness landscape (Figure 1A). In the opposite, high mutation rate extreme, the evolutionary process is defined by clonal interference: the population consists of multiple competing clones that differ from each other by several mutations (Fogle et al., 2008; Keller et al., 2012). In this limit, the population explores multiple trajectories in parallel so that evolution can be represented by a time-dependent distribution in sequence space (Figure 1B). To decouple the effect of the landscape topography on the predictability of evolution from the intricacies of the evolutionary dynamics, researchers often assume the strong selection, weak mutation (SSWM) limit in which mutations are fixed sequentially (sweep dynamics) and the probability of fixation of a deleterious or neutral mutation is essentially nil (no genetic drift; Gillespie, 1985; Slatkin and Muirhead, 1999). Evolutionary paths in the SSWM limit are monotonic trajectories on the fitness landscape (Figure 2A). Thus, when selection is strong and mutation rate is low, the statistics of monotonic paths quantify the accessibility of high fitness peaks and, to some degree, the predictability of mutational trajectories during adaptation. Peak accessibility is usually defined as the probability of finding at least one monotonic path to the highest peak averaged over the starting point on and/or over the statistical ensemble of model landscapes. Another frequently measured quantity is the distribution of the number of monotonic paths to the main summit. This quantity is frequently regarded as the indicator of repeatability of evolution or predictability of evolutionary trajectories: the greater the mean number of monotonic trajectories to the peak, the less predictable evolution is thought to be (Weinreich et al., 2006; Poelwijk et al., 2007; O’Maille et al., 2008; Reetz and Sanchis, 2008).

Indeed, there should be a negative correlation between the number of monotonic paths and the predictability of the trajectory that is realized. However, the notion of predictability can be made more precise by quantifying the degree of similarity between the monotonic paths. The idea is that a large number of similar, clustered paths represents a high degree of repeatability whereas a small number of dissimilar paths signifies low predictability. Accordingly, we recently introduced the concept of mean path divergence to quantify evolutionary predictability (Lobkovsky et al., 2011).

Predictability is defined for an ensemble of trajectories. Each trajectory must have an associated probability of occurrence under the specified evolutionary dynamics. In the SSWM limit all beneficial mutations are fixed whereas all neutral and deleterious mutations are purged. Thus, the probability of fixing a particular beneficial mutation is simply the inverse of the number of available mutations that increase fitness at that point on the landscape. The probability of occurrence of a monotonic path in the SSWM limit is therefore the inverse of the product of the number of possible beneficial mutations at every point along the path. The ensemble path divergence is defined as the sum over all pairs of paths of the product of the probabilities of their occurrence and the inter-path distance (see Figure 2 for more detail). The distance between paths can be defined in a number of different ways. A natural measure of the inter-path distance is the sum of the minimum Hamming distances from each point on one path to the other path divided by the combined total length of the two paths (see Figure 2B for more detail).

Having defined the path divergence of an ensemble of paths, we quantify the predictability of evolution on a particular landscape by computing the mean path divergence among ensembles of monotonic paths that originate at all possible points on the landscape and terminate at one the of the peaks.

We used the path divergence, statistics of monotonic paths, and several measures of landscape roughness to quantify the predictability of evolution in three classes of fitness landscapes: (1) noisy additive landscapes (see Box 1 for more detail), (2) currently available complete empirical fitness landscapes (see **Box 1**), and (3) landscapes derived from a model of heteropolymers in which fitness was equated with the robustness to misfolding (Lobkovsky et al., 2010, 2011). Our goal was to examine the association between roughness and evolutionary predictability. It is impossible to capture the roughness of a multidimensional landscape in a single parameter. We therefore utilized four distinct measures of roughness summarized in Box 1. We showed that empirical and model-derived landscapes were significantly smoother than their randomly permuted counterparts. The roughness measures were found to be good proxies for evolutionary predictability. The roughness measures as well as the measures of evolutionary predictability of empirical and folding model-derived landscapes were consistent with those of moderately perturbed additive landscapes. The model landscapes exhibited a deficit of suboptimal peaks even compared with noisy additive landscapes with similar overall roughness. We conjectured that the relative smoothness, mutational robustness, and substantial deficit of peaks in fitness landscapes of protein evolution are the consequences of the fundamental physics of protein folding.

Box 1 **Smooth and rough fitness landscapes.**

**Noisy Additive Landscapes**

An instance of an additive landscape perturbed by noise was constructed by first assigning a fitness effect drawn from an exponential distribution to every substitution in the peak sequence. To obtain the fitness of a mutant sequence, we subtract the sum of the fitness effects of mutations from the peak fitness. This quantity was modified by multiplicative noise via a multiplication a number drawn from a uniform distribution [0,1] raised to a positive power. When this power is small, multiplicative factors are close to unity and the perturbation is small as well. If the perturbed fitness was positive, the mutant was included into the landscape. The noise amplitude was varied to obtain a family of landscapes of continuously varying roughness.

**Empirical Landscapes**

In Lobkovsky et al. (2011) we analyzed two exhaustively sampled empirical landscapes: (1) five substitutions in TEM *β*-lactamase conferring antibiotic resistance (Weinreich et al., 2006); and (2) eight substitutions in Sesquiterpene synthase which toggle the major reaction product (O’Maille et al., 2008).

**Measures of Roughness**

We used four measures of roughness: (1) deviation from additivity (Carneiro and Hartl, 2010); (2) root mean squared difference between the fitness of a point and its neighbors, averaged over the entire landscape; (3) the number of points with no fitter neighbors divided by the total number of points in the landscape (peak fraction); and (4) mean distance to the tree component which is a set of points that have at most a single fitter neighbor.

## 4 Concluding Remarks

Clearly, quantitative study of evolutionary trajectories is still in its infancy. Parallel evolution experiments directly probe predictability but face a number of daunting technical challenges such as the difficulty of maintaining uniformity of the environment, and tracking and sequencing a large number of populations and individuals. Therefore important complementary approaches involve empirical characterization of fitness landscapes and their theoretical analysis in terms of evolutionary characteristics such as peak accessibility and predictability of adaptation.

Currently, to the best of our knowledge, due to technical challenges of isolating all combinatorial mutants and measuring their fitness, complete empirical fitness landscapes include combinations of at most eight binary substitutions. In other words, these evolutionary experiments explore only a minuscule fraction of the sequence space. As the available empirical fitness landscapes become more numerous and larger, their continuous analysis will shed further light on the extent of variation of evolutionary predictability and its dependence on the landscape size and structure. Other types of landscapes such as the aptamer binding affinity (Warren et al., 2006; Knight et al., 2009) or RNA folding energy (Cowperthwaite et al., 2008) landscapes do not require special fitness assays and therefore can be substantially larger. Analysis of the roughness measures and evolutionary predictability for these landscapes can determine whether, like the fitness landscapes, the DNA binding and RNA folding landscapes can be approximated by moderately perturbed additive landscapes. The analysis of larger landscapes will also shed light on the dependency of evolutionary predictability on the landscape size.

Another potentially fruitful avenue of investigation is a thorough analysis of other types of model landscapes in addition to noisy additive landscapes. Franke et al. (2011) computed the statistics of monotonic paths for several classes of model landscapes including uncorrelated landscapes, rough mount Fuji landscapes (Aita et al., 2000), the *NK* model landscapes (Kauffman and Weinberger, 1989), and the neutral landscapes with randomly inserted inaccessible islands. An empirical landscape of dimensionality 8 (de Visser et al., 2009) was found to be similar to a moderately correlated case of the *NK* model, and peak accessibility as well as the number of alternative trajectories to the peak were both found to increase with the landscape size. Further analysis of the broad classes of model landscapes as well as emerging empirical landscapes, in particular calculation of the path divergence and roughness measures, are expected to help with the classification of landscapes and illuminate the nature of the connections between roughness, size, and evolutionary predictability. Finally, if the assumption of stationarity of the fitness landscape were relaxed, the landscape would become a seascape (Mustonen and Lassig, 2010). The peak location would be no longer fixed, and therefore our formulation would have to be modified to include an appropriate averaging procedure.

It is far premature to attempt a general conclusion on the degree of repeatability of evolutionary trajectories. Moreover, this degree critically depends on the specifics such as the number of loci involved in adaptation, the existence of alternative adaptation pathways, and the environmental (or experimental) conditions so that a general solution simply might not exist. All these caveats notwithstanding, recent experimental and model studies make it abundantly clear that short-term evolution in a fixed environment is far more predictable in a quantitative sense measured by the path divergence than it would be in an uncorrelated landscape. In other words, although multiple evolutionary trajectories are often accessible, evolution is strongly constrained and the part of the fitness landscape available for exploration is highly variable but typically small. Thus, if we actually could replay the tape of evolution, the outcome could have been considerably more similar to the existing diversity of life forms than Gould expected.

## Conflict of Interest Statement

The authors declare that the research was conducted in the absence of any commercial or financial relationships that could be construed as a potential conflict of interest.
